# Symptom Covering

**Published:** 1884-01

**Authors:** 


### HOMŒOPATHIC PHYSICIAN.

A MONTHLY JOURNAL OF MEDICAL SCIENCE.

"If our school ever gives up the strict inductive method of Hahnemann, we are lost, and deserve only to be mentioned as a caricature in the history of medicine."—CONSTANTINE HERING.

Vol. IV. JANUARY, 1884. No. 1.

### EDITORIAL.

SYMPTOM COVERING.—The practice of Homœopathy is comprised in the words *symptom covering*—nothing more, nothing less. *But symptom covering* means a great deal, and what at first sight would appear an easy, childish task, on further examination, and when carried out to its fullest, broadest limits, becomes an herculean task—one which requires the keenest observation, the nicest judgment, the best-educated mind of any work we know of. This can be easily illustrated. Suppose a case is before you for treatment: Is your duty fully performed by merely asking a few general questions, and then carelessly prescribing a remedy? This is very often done, and failure is the result. But the physician who *tries* to practice Homœopathy performs his duty in a much more thorough manner. He examines his patient most carefully; every symptom is noted down, and each is fully inquired into. Nausea is complained of. Shall that pass as a symptom? Never; it is of no value. What is the character of the nausea? When felt? What causes or relieves it? These questions being answered, the combined symptom becomes of value. The patient is constipated. Is constipation a symptom of value? Not at all; all its conditions, peculiarities, etc., must be known before it can be any assistance in prescribing. So we learn that *each* symptom must be studied and investigated. But, to be brief, let us suppose the proper remedy to be found. The next question, is, How shall it be given? How often and in what potency? The nicest judgment is needed to correctly solve this knotty problem. Experience alone can furnish the ability to do it successfully. As a general rule, it may be said that it is best to give a high potency (say thirty or two hundred) in *one dose* for the first prescription. If that fails to do good (symptoms being the same), then go higher and try more frequent doses. A few doses in water, given one, two, or three hours apart, for, say, a half dozen times, will often be found of service. In acute attacks, occurring in patients of general good constitution, it is simply astounding to observe the effects of this *one dose*. No one will or can credit the power of this single minute dose *until he has tried it*. This much may be safely relied on: in repeating your medicines, *look at the patient, not at the clock or calendar!* It is stupid to say, as some do, In acute cases repeat every few minutes; in chronic, every few days, etc. In neither acute nor in chronic cases will the wise doctor repeat the remedy while the case is improving, no matter whether the last dose was given ten minutes since, or ten days, or ten weeks ago. The patient is improving. What more can be secured? A faster recovery? Nature generally selects the proper pace, and your attempts at spurring her on can only be injurious.

But it may be asked, How is one to know if the patient is improving? Here pathology comes into service. The physician should be able to properly name, to catalogue, so to speak, the disease. This is important, as it enables one to know the general course, the clinical history of the case before him. Knowing, then, the course which such cases generally run, he can readily judge if the changes in the case before him indicate improvement or the reverse. If improvement be evident, one's duty is clear: let the patient alone. But suppose the symptoms indicate aggravation, what then? Is the aggravation due to the remedy—this will rarely be the case if only one dose be given at first, as a "feeler"—or is it due to the disease? Such questions arise every day, and upon their correct solution depend the patient's life, the physician's reputation. This difficulty can only be met satisfactorily in each case by a careful comparison of the patient's symptoms and those of the disease from which he suffers in general. If the aggravation follows immediately, and somewhat suddenly, upon the giving of the medicine, it would be safe to wait before doing anything. On the other hand, if it appears to be such as is common in the disease, it would be better to repeat the remedy, or to change, if symptoms clearly indicate another medicine.

It is well to think over the cases one is about to visit during the day, to decide in one's mind as to the condition you expect to find them. Thus, Mr. A, yesterday, was so-and-so. If my medicine has done its work, he should to-day be in such-and-such a condition; if my prescription was faulty, and the disease is running its common course, he will present such another condition. This mental review of cases can be easily done as one goes from house to house, and will be found of much service. The sick-room, with its confusion, anxieties, and questioning friends, is a poor place for careful reflection.

To recapitulate, in symptom-covering the following require careful attention:

First, the thorough examination of the patient to acquire the basis of the prescription.

Secondly, the selection of a drug upon the symptoms gathered.

Thirdly, the choice of potency, the decision as to the frequency of the dose.

Fourthly, the changes occurring in the patient; do they indicate improvement, or the reverse? shall it be Sac lac, a repetition of the remedy, or a new selection?

Fifthly, in chronic cases the presence of a chronic miasm, psora, syphilis, etc., must be ascertained.

These few sentences give but a faint idea of the many difficulties to be met with in the practice of Homœopathy. The work is most arduous and exacting, but well performed, the reward is great. Only to those who have never tried this method thoroughly does it seem easy or foolish or unscientific. The greater the mind, the riper the judgment, so much the greater and grander will the success be. Success in homœopathic practice comes not often to the dolt nor to the sluggard.

